# Usual Intake of Flavonoids Is Inversely Associated with Metabolic Syndrome in African American and White Males but Not Females in Baltimore City, Maryland, USA

**DOI:** 10.3390/nu14091924

**Published:** 2022-05-04

**Authors:** Rhonda S. Sebastian, Marie T. Fanelli Kuczmarski, Joseph D. Goldman, Alanna J. Moshfegh, Alan B. Zonderman, Michele K. Evans

**Affiliations:** 1 U.S. Department of Agriculture, Agricultural Research Service, Beltsville Human Nutrition Research Center, Food Surveys Research Group, 10300 Baltimore Avenue, Building 005, Beltsville, MD 20705-2350, USA; joe.goldman@usda.gov (J.D.G.); alanna.moshfegh@usda.gov (A.J.M.); 2 National Institutes of Health, National Institute on Aging, Laboratory of Epidemiology & Population Sciences, 251 Bayview Boulevard, Baltimore, MD 21224, USA; zondermana@gmail.com (A.B.Z.); evansm@grc.nia.nih.gov (M.K.E.)

**Keywords:** flavonoids, usual intakes, metabolic syndrome, racial differences, diet, Healthy Aging in Neighborhoods of Diversity across the Life Span (HANDLS) study

## Abstract

Despite research that suggests flavonoids protect against metabolic syndrome (MetS) and evidence that intake of these compounds differs by race, knowledge about whether flavonoid–MetS associations vary among racial groups is limited. This study sought to estimate usual total flavonoid intake in African American and White adults and assess its sex- and sex/race-specific associations with MetS and its risk factors. Analysis of cross-sectional data from 1837 adults participating in the Healthy Aging in Neighborhoods of Diversity across the Life Span (HANDLS) study were analyzed. Usual total flavonoid intake was estimated using the NCI Method, and logistic regression measured its linkages with health outcomes. Among males overall and when stratified by race, odds of MetS and its risk factors low high-density lipoprotein cholesterol (HDL-C) and elevated glucose were lower at the 75th percentile of usual total flavonoid intake than at the 25th percentile (OR for MetS = 0.62; 95% CI = 0.53, 0.71). However, low HDL-C and elevated glucose were positively associated with usual flavonoid intake among females. The comparable associations by race within sex imply that the relationships between flavonoid and health outcomes may be evident across an array of intakes.

## 1. Introduction

Metabolic syndrome (MetS) prevalence in the United States is high, impacting 36.9% of adults aged ≥20 years and 50.4% of those aged ≥60 years in 2015–2016 [1]. It substantially increases the likelihood of negative health outcomes, including heart disease and diabetes [2]. Individuals are considered to have MetS when three or more of the following risk factors are present: central obesity, elevated blood pressure, elevated blood glucose, elevated triglycerides, and low high-density lipoprotein cholesterol (HDL-C) [2].

Dietary intake is widely acknowledged to influence the development of MetS. Diets associated with reduced levels of MetS and its risk factors emphasize intake of fruits, vegetables, whole grains, nuts, and legumes [3,4,5,6]. Flavonoids, which are polyphenolic compounds found in plants, may partially explain the apparent benefit of some of these foods in preventing MetS. Flavonoids have well-documented anti-inflammatory, antioxidant, and other favorable effects on multiple body systems that could be relevant to MetS [7,8,9,10]. For example, flavonoids reduce lipid absorption [7,9,10] and lipogenesis [10], potentially affecting blood lipid levels. They have been shown to improve endothelial function and nitric oxide bioavailability [7,10,11], which could lower blood pressure. Flavonoids can also modulate enzymes involved in glucose transport and insulin signaling [7,8,10,12], which could mitigate insulin resistance and subsequent elevations in fasting blood glucose.

When investigating associations between diet and health outcomes, estimates of long-term dietary exposures are required. The National Cancer Institute (NCI) has developed a method that models aspects of dietary intake derived from 24 h recalls (24HR) to allow estimation of long-term average, or “usual”, intakes of nutrients, foods, or food components for a given population [13,14]. The NCI method is designed to reduce bias in the assessment of dietary intake by addressing measurement error issues inherent in 24-h recalls (24HR), including but not limited to the excess intra-individual variation reflected in estimates derived from a single day of dietary intake [14]. Statistical techniques applied to usual intake estimates allow for the assessment of error-corrected associations between usual intake and health outcomes [15].

Mean estimated flavonoid intakes have been shown to be lower among African American adults as compared to White adults [16]. However, it is unknown if the association between flavonoid intake and health outcomes such as MetS varies by race. It is possible that, because of lower intakes, the benefits attributable to flavonoids on MetS and its constituent risk factors may be less evident among African American adults. For this reason, the objectives of the current study were to estimate usual intake of total flavonoids using the NCI method in an African American and White adult urban population and to identify its associations with MetS and its constituent risk factors in these racial groups.

## 2. Materials and Methods

### 2.1. Study Sample

Healthy Aging in Neighborhoods of Diversity across the Life Span (HANDLS) is a prospective cohort study conducted in Baltimore City, MD. The HANDLS sample design [17,18] incorporated a 4-way stratification by age (seven 5-year age groups, 30–64 years), sex, race (African American and White), and income operationalized as poverty status (self-reported household income above or below 125% of the 2004 Health and Human Services poverty guidelines) [19]. Participants were recruited from an area probability sample composed of 13 neighborhoods (groups of contiguous census tracts) that were selected based on the likelihood of enlisting men and women in the target racial, poverty status, and age groups. Pregnancy and active cancer treatment within the last 6 months were exclusion criteria [18]. Details regarding the HANDLS sample, design, and methods are available elsewhere [17,18,20].

Data collection occurred in two phases [17,18]. Phase 1 took place at each participant’s home, and Phase 2 took place in the study’s mobile research vehicles (MRVs). Information collected during the home visit included educational level, family income level, health status, and one 24HR per participant. The Phase 2 in-depth examination included a medical history (including questions related to smoking, menstrual status, and use of medications), physical examination, and fasting blood draw. Additional Phase 2 data included a second 24HR and an assessment of literacy level using the reading subtest of the Wide Range Achievement Test-3rd Edition (WRAT-3) [21].

**Figure 1:**
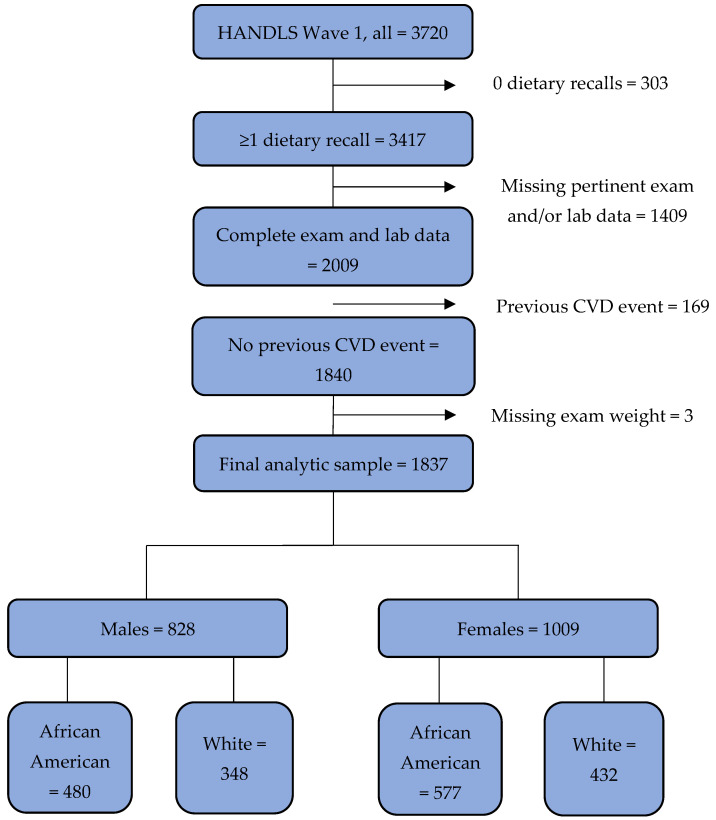
Flow diagram illustrating criteria for inclusion in study sample and analytic subgroups and counts. Abbreviations: HANDLS, Healthy Aging in Neighborhoods of Diversity Across the Life Span study; CVD, cardiovascular disease.

This study was a cross-sectional analysis of Wave 1 data, which were collected from 2004 through 2009 [18]. The Wave 1 sample consisted of 3720 participants (Figure 1). Over 90 percent of participants provided one 24HR, and 84–85% in each sex and sex/race group provided two 24HRs. Of the 3417 participants who completed at least one 24HR, 2009 visited the MRV and had complete data on all pertinent exam and laboratory measures.

After exclusion of an additional 169 individuals due to a previous cardiovascular event and 3 due to missing exam weights, the final analytic sample consisted of 1837 individuals (828 males; 1009 females).

### 2.2. Flavonoid Intake Assessment

The 24HRs in HANDLS were conducted by trained interviewers using the U.S. Department of Agriculture (USDA) Automated Multiple-Pass Method [22,23]. Measurement aids facilitated accurate estimation of quantities of foods and beverages consumed [17]. After collection, quality control procedures developed by the USDA were conducted to verify the completeness and plausibility of the 24HR data.

All foods and beverages were coded using the USDA Food and Nutrient Database for Dietary Studies (FNDDS) [24]. Each food/beverage was then linked to flavonoid values from the Database of Flavonoid Values for USDA Survey Food Codes 2007–2010 (Flavonoid Database) [25,26] by means of the USDA food code. Although the Flavonoid Database was designed primarily to be used with WWEIA, NHANES data, it can also be applied to data from other studies [27]. The development of the Flavonoid Database has been described previously [28]. Briefly, for each food/beverage in FNDDS, the Flavonoid Database provides the amounts (mg/100 g) of 29 common flavonoids in 6 flavonoid classes (anthocyanidins, flavan-3-ols, flavanones, flavones, flavonols, and isoflavones) [26]. Flavan-3-ols includes catechins, theaflavins, and thearubigins. In the present study, total flavonoid intake was defined as the sum of the 6 classes in the Flavonoid Database.

All food/beverage items reported in HANDLS were included in calculating flavonoid intakes. However, since information on supplement use was not collected in Wave 1, contributions from dietary supplements were not assessed.

### 2.3. Metabolic Syndrome and Its Risk Factors

For this study, participants were considered to have MetS if at least three of the following five risk factors were present: (1) central obesity, (2) elevated blood pressure, (3) elevated glucose, (4) elevated triglycerides, and 5) low HDL-C [2]. Risk factors were assessed on the basis of the “harmonized” MetS diagnostic criteria [2]. Central obesity was assessed using U.S.-specific thresholds for waist circumference (WC): ≥102 cm for males; ≥88 cm for females. The criteria for elevated blood pressure were systolic blood pressure ≥130 mm Hg and/or diastolic blood pressure ≥85 mm Hg; for elevated glucose, a fasting value of ≥100 mg/dl (>5.6 mmol/L); for elevated triglycerides, ≥150 mg/dl (1.7 mmol/L); and for low HDL-C, <40 mg/dL (<1.0 mmol/L) for males and <50 mg/dl (<1.3 mm/L) for females. Participants who were currently taking prescription medication(s) to address blood pressure or any of the other blood measures were considered to have those risk factors regardless of their exam/laboratory values.

Exam and laboratory data were collected by a physician or nurse practitioner following standardized procedures. Body mass index (BMI) was calculated from measured height and weight [29]. To determine WC, a tape measure was placed beginning at the hip bone and wrapped around the waist at the level of the navel [29]. Blood pressure was measured in each arm using the brachial artery auscultatory method [29]. For both systolic and diastolic measurements, readings from the left and right arm were averaged prior to analysis. Glucose, triglycerides, and HDL-C were measured in whole blood collected after participants fasted for a minimum of 8 h, and assessments were performed using a spectrophotometer (AU5400 Immuno Chemistry Analyzer; Olympus, Center Valley, PA, USA) [29].

### 2.4. Statistical Analysis

SAS^®^ 9.4 (2012, SAS Institute Inc., Cary, NC, USA) and SAS^®^/STAT 14.2 (2016, SAS Institute Inc., Cary, NC, USA) were used in data preparation and analysis. Both SAS-callable SUDAAN^®^, release 11.0 (2012, RTI International, Research Triangle Park, NC, USA), employing a Taylor series method for characteristics of the sample and SAS code employing a balanced repeated replicate (BRR) method for analyses involving usual intake were used to account for the HANDLS sample design in variance estimation and statistical testing. Sample weights provided with the HANDLS data were recalibrated to adjust for the subsetting of the original analytic sample and applied in all analyses to permit calculation of estimates representative of the Baltimore City adult population with the targeted demographics. The replicate weights associated with the BRR method were generated from this subset of the sample weights and calibrated to the same population targets as the original full-sample weights, that is, the adult African American and White population of Baltimore City.

Percentages of the HANDLS population by category of selected demographic, lifestyle, and health-related variables for males, females, and by race within sex were estimated. Demographic characteristics were age (in years), income as poverty status (above or below 125% of the 2004 HHS Poverty Guidelines) [19], education (<high school diploma/GED, high school diploma/GED, post-secondary education), and literacy level (≤8th grade, >8th grade). Smoking status (currently smoking, not currently smoking) was the only lifestyle characteristic examined. Health-related variables were categorized as self-reported and diagnostic. Self-reported health measures were health status (excellent/very good, good, fair/poor), and menopause status (women only: pre-menopausal, post-menopausal). Diagnostic health measures were BMI and prevalence of MetS and its 5 risk factors.

Usual intake means, standard errors, and percentiles of total flavonoids by sex and by race within sex were estimated via the NCI method [14] implemented with SAS programming. Briefly, NCI’s MIXTRAN macro was first used to fit a statistical model representing the usual intake distribution and, secondly, the DISTRIB macro was used to randomly draw 100 simulated intakes tied to each person in the sample through characteristics of interest, namely, race, income and age [15]. The point estimates of usual intake were then constructed from the simulated intakes. The process was repeated multiple times utilizing a BRR method to estimate variances.

Generally, the fitting of the statistical model is a two-step process where models of the probability of consumption and the amount consumed are fitted simultaneously and

Usual intake=Probability ∗ Amount

However, as total flavonoids are consumed by nearly everyone on any day, in this situation the probability of consumption is assumed to be one, and a model for the probability of consumption does not need to be fitted. Note that the amount model is fitted under a transformed scale. The estimation process based on the simulated intakes includes a back-transformation to the original scale.

The linkages of health outcomes with usual intake of flavonoids (as represented by odds ratios) were estimated via application of the NCI method’s INDIVINT macro for prediction of individual food or nutrient intake for use in a disease model [15]. This process utilizes the statistical model representing the usual intake distribution. From this fitted distribution, each individual’s usual intake is predicted through regression calibration [30]. These predicted usual intakes under the transformed scale are used only to remove bias from estimated diet–outcome associations and cannot be used to classify individuals into flavonoid intake categories, but they may be used as regressors in logistic or linear regression models. In this study, a logistic regression model predicting an outcome from the regression-calibrated usual intake along with selected covariates (i.e., demographic and lifestyle characteristics and self-reported health measures previously described and BMI) was fitted. The 25th and 75th percentiles of the sex-specific and sex-and-race-specific usual intake distributions in the transformed scale were utilized to produce odds ratios. BRR was utilized for the construction of 95% confidence intervals for the odds ratios.

A type I error rate of *p* < 0.05 was applied in all analyses.

## 3. Results

### 3.1. Population Characteristics

Demographic, lifestyle, and health parameters for the HANDLS population by sex and by race within sex are described in Table 1. These percentages are based on counts weighted to reflect the Baltimore City African American and White adult population.

Age and poverty status are among the variables integral to the HANDLS study design, and they were adjusted in the calculation of sample weights. The remaining demographic variables revealed that 24.3% of males and 22.4% of females had an educational attainment less than a high school diploma or GED, and 36.6% of males and 29.1% of females had a literacy level at or below 8th grade. Self-reported lifestyle and health measures indicated that 49.8% of males and 35.8% of females were current smokers, and approximately 1 out of 5 persons reported their health status as fair or poor.

The prevalence of MetS was 28.2% among males and 37.9% among females. Among White males, African American females, and White females, over one-third met the MetS criteria; among African American males, 23.1% did so. In every sex- and sex/race group examined, over one-fourth presented with the MetS risk factors low HDL-C, elevated blood glucose, and elevated waist circumference. The prevalence of elevated blood pressure was ≥40.2% in both sexes and in every sex/race group except White females. Elevated waist circumference was observed in 71.0% of females overall, and 77.3% and 60.0% of African American and White females, respectively.

### 3.2. Usual Total Flavonoid Intake Distributions

As shown in Table 2, estimated mean usual intake of total flavonoids was 187.4 ± 10.6 mg/d for all males and 255.2 ± 34.6 mg/d for all females. The median intakes (50th percentile) for these same population groups, 126.3 ± 8.9 mg/d for males and 149.8 ± 19.3 mg/d for females, were lower than the corresponding means, indicating that the flavonoid intake distributions were positively skewed (i.e., there was a preponderance of low intakes). This finding was consistent across all sex/race groups. Usual intakes of total flavonoids at 6 additional percentiles, including the 25th and 75th percentiles, are also presented in Table 2.

### 3.3. Flavonoid Intake—MetS Associations

Among all males, the probability of having MetS was 38% lower at the 75th percentile of usual total flavonoid intake than at the 25th percentile (OR = 0.62; 95% CI = 0.53, 0.71) (Table 3). Odds did not vary between African American males (OR = 0.72; 95% CI = 0.53, 0.98) and White males (OR = 0.72; 95% CI = 0.52, 0.98).

The likelihood of meeting the diagnostic criteria for two of the individual MetS risk factors was also inversely associated with usual total flavonoid intake among males. Odds of having a low HDL-C level were 48% lower at the 75th percentile of usual flavonoid intake than at the 25th percentile (OR = 0.52; 95% CI = 0.45, 0.61). Comparable findings were observed by racial group. Among African American males, those at the 75th percentile were 42% less likely to have low HDL-C than were those at the 25th percentile of usual total flavonoid intake (OR = 0.58; 95% CI = 0.42, 0.81). The odds were similar for White males (OR = 0.57; 95% CI = 0.41, 0.81), even though the usual total flavonoid intake distribution, and thus the 75th and 25th percentile values, were different. Similarly, the odds of elevated blood glucose levels were 21% lower at the 75th percentile of usual flavonoid intake than at the 25th percentile among males overall (OR = 0.79; 95% CI = 0.69, 0.89), with analogous findings by race. Odds of having elevated triglycerides, blood pressure, and waist circumference were not associated with usual total flavonoid intake among males overall or in either racial group.

Among females, the odds of having MetS did not differ between the 75th percentile and the 25th percentile of the usual total flavonoid distribution (OR = 1.22; 95% CI = 0.92, 1.61). Findings among African American females and White females were consistent with the results observed for females overall.

The only significant findings regarding females were contrary to those for males. The odds of low HDL-C at the 75th percentile of usual total flavonoid intake relative to the 25th percentile were 34–36% higher among females overall (OR = 1.34; 95% CI = 1.07, 1.69) and when stratified by race (African American, OR = 1.36; 95% CI = 1.02, 1.82; White, OR = 1.35; 95% CI = 1.02, 1.79). Similarly, the prevalence of elevated glucose was positively associated with usual flavonoid intake among all females (OR = 1.61; 95% CI = 1.18, 2.18), African American females (OR = 1.57; 95% CI = 1.20, 2.06) and White females (OR = 1.56; 95% CI = 1.19, 2.04).

## 4. Discussion

Numerous investigations have focused on the relationship between flavonoid intakes and health outcomes, but none have analyzed associations between usual total flavonoid intake as derived from 24HRs and MetS and its risk factors by both sex and by racial group within sex as was done in this study. For all health outcomes analyzed, associations did not vary by race among either males or females. However, a major finding was the lower odds of MetS at the 75th percentile of usual total flavonoid intake than at the 25th percentile among males. Among females, no association between usual flavonoid intake and MetS was observed.

Multiple studies have reported associations between flavonoid intake and an individual MetS risk factor (e.g., hypertension), but fewer analyzed MetS specifically. In a cross-sectional study of Iranian adults, Sohrab et al. reported inverse associations between total flavonoid intake assessed via food frequency questionnaire (FFQ) and prevalence of MetS, elevated WC, fasting glucose, triglycerides, and blood pressure [31]. Similar results were found by Qu et al. among Chinese adults, with those in the highest quartile of FFQ-estimated total flavonoid intake displaying a 23% reduction in odds of MetS compared to those in the lowest quartile [32]. Neither of these studies investigated associations stratified by sex. An inverse association between MetS and 24HR-assessed intake of flavan-3-ols, the largest flavonoid class contributor to total intake in the U.S. and elsewhere [28,33,34], was found in the Korean National Health and Nutrition Examination Survey among females but not males [35]. In contrast, in a sample of 8821 Polish adults, Grosso et al. found no association between flavonoid intake assessed via FFQ and MetS, though that intake was associated with lower odds of impaired fasting blood glucose [36].

Only two studies have been identified that analyzed associations between usual flavonoid intake as derived from 24HRs and one of the MetS risk factors [37,38], and only one of those investigated total flavonoid intake. After estimating usual intake of flavonoids and other polyphenols via the multiple source method [39,40], Miranda et al. analyzed its associations with hypertension among 550 Brazilian adults [38]. Flavonoid classes included in the total were not specified. Median usual intake of total flavonoids was 34.5 (interquartile range: 17.4, 61.6) mg/d, or approximately one-fourth of that reported in this study. Whereas intake of other polyphenol classes was inversely associated with hypertension prevalence, flavonoid intake was not; the latter finding was echoed in the current study.

None of these studies, regardless of flavonoid assessment method (i.e., FFQ, one or more 24HRs, or usual intake), analyzed differential associations by race. Instead, populations examined were either fairly homogenous with regards to racial group [31,32,35], or the race of participants was not specified [36,38].

An inverse association between usual total flavonoid intake and low HDL-C was observed among HANDLS study males. This finding aligns with previous investigations that reported positive associations between intake of total flavonoids or flavonoid classes and HDL-C levels [33,41]. Some recent research suggests that the beneficial effect of flavonoids on HDL-C may involve mechanisms beyond simply increasing its concentration, though those were not analyzed in this study [42,43]. Similarly, the inverse association observed between usual total flavonoid intake and elevated blood glucose among males in the HANDLS population concurs with the conclusion of a meta-analysis that found flavonoid intake is related to a reduced risk of Type 2 diabetes [44]. In all, this study’s findings suggest that, for males, interventions to promote inclusion of flavonoid-rich foods and beverages in the diet could be a useful complement to treatments for mitigation of these risk factors.

Paradoxically, among females, usual flavonoid intake was detrimentally associated with the same risk factors for which beneficial associations were observed in males. The positive relationships between usual flavonoid intake and low HDL-C and elevated glucose among females are difficult to explain. To our knowledge, these contrary associations by sex have not been reported in the literature. HANDLS represents an exclusively urban, historically underserved population [17]. Its members exhibit many characteristics that increase their likelihood of MetS and its risk factors, including higher than national percentages who are current smokers, have very low family incomes, low education level, and fair/poor self-perceived health status [45,46,47,48,49]. Though these variables were included in the model, it is possible that other relevant factors that differed by sex were not considered. Investigation of other dietary factors related to these outcomes, e.g., added sugar intake, did not show differences in intake between males and females. Previous investigations of the HANDL population have found that systemic inflammation as measured by biomarkers is significantly higher in females as compared to males, though the reasons for that disparity are not clear [50,51]. It may be that flavonoid intake was not sufficient to counteract the causes of higher inflammation experienced by females. In any case, repeating this analysis in a nationally representative sample of African American and White adults could inform whether these conflicting findings by sex are generalizable beyond the unique population the HANDLS study represents.

Usual total flavonoid intake distributions varied by race within sex, and thus usual intakes at the contrasted percentiles (75th, 25th) did so as well. Nevertheless, odds for each outcome were very similar between African American and White individuals within sex. These findings of comparable associations across a range of intakes have implications for the currently evolving process of quantifying intake recommendations for these food components [52]. Additional perspective is provided by previously published research that reported that flavonoid intakes in the HANDLS population were significantly below those of the U.S. population [53], implying that intake thresholds required to observe flavonoid–health associations may be relatively low.

This investigation has a number of strengths, principally due to the sample, the application of the usual intake methodology, and the flavonoid database. The stratified design of the HANDLS study permits explorations of differential associations between variables such as diet and health outcomes among sociodemographic groups. In this study, the relatively large number of African American participants allowed investigation of associations by race. Moreover, use of available study weights enabled extrapolation of these findings to the African American and White adult population of Baltimore City. Application of the NCI method to assess usual total flavonoid intakes greatly enhances the validity of reported flavonoid–MetS associations. Correcting (to the extent possible) for the measurement error caused by intra-individual variation leads to less biased estimates and prevents a loss of statistical power that could invalidate statistical testing used in regression analyses assessing diet–health outcomes [54]. Finally, the flavonoid database employed in this study is comprehensive; it contains values for total flavonoids in six classes for over 7000 foods and beverages and includes the contribution of flavonoid-containing ingredients in multi-ingredient foods [26]. Consequently, it was possible to assign appropriate flavonoid profiles to all foods and beverages reported in HANDLS.

This study has a few limitations. First, because the NCI methodology does not classify individuals into categories, prevalence estimates for MetS and its risk factors corresponding to the percentiles of usual total flavonoid intake could not be determined. Second, the 24HRs from which the usual intakes are modeled were self-reported. Even though the NCI method addresses some sources of measurement error, it cannot correct for respondent bias caused by misreporting. However, the dietary data collection method, the automated multiple-pass method, has been validated using the doubly labeled water technique and shown to reduce bias in the collection of energy intakes [22]. Third, multiple significance tests were conducted, which increased the likelihood of Type I error. Fourth, flavonoid intake from dietary supplements was not assessed, as these data were not collected in Wave 1. Inclusion of supplement intake could impact observed flavonoid–outcome associations. Finally, only total flavonoids were analyzed in this study. Associations between usual intakes of specific flavonoid classes and MetS and its risk factors would likely differ.

## 5. Conclusions

In this at-risk population of African American and White adults in Baltimore City, usual total flavonoid intake was inversely associated with MetS and its risk factors low HDL-C and elevated fasting blood glucose in males. Conversely, usual flavonoid intake was positively associated with low HDL-C and elevated fasting blood glucose among females. Relationships did not differ by race within sex despite differing intake distributions, suggesting that flavonoid–health outcome associations may be evident across a range of intakes.

## Figures and Tables

**Table 1:** Characteristics ^1^ of individuals by race within sex, Healthy Aging in Neighborhoods of Diversity across the Life Span (HANDLS) study, Wave 1 ^2^.

	Males			Females		
Characteristic	All	African American	White	All	African American	White
Demographic:						
Age ^3^, y, % (SE)						
30–49	60.3 (1.7)	59.3 (1.8)	62.0 (1.4)	60.3 (3.7)	60.3 (5.3)	60.2 (2.3)
50–64	39.7 (1.7)	40.7 (1.8)	38.0 (1.4)	39.7 (3.7)	39.7 (5.3)	39.8 (2.3)
Poverty status ^3,4^, % (SE)						
<125% poverty	16.7 (3.0)	19.5 (4.4)	11.6 (1.7)	22.8 (1.7)	28.1 (2.6)	13.4 (2.3)
>125% poverty	83.3 (3.0)	80.5 (4.4)	88.4 (1.7)	77.2 (1.7)	71.9 (2.6)	86.6 (2.3)
Education, % (SE)						
<High school diploma/GED	24.3 (3.6)	28.3 (3.2)	17.0 (7.3)	22.4 (2.5)	25.1 (3.5)	17.6 (6.2)
High school diploma/GED	31.9 (2.1)	37.0 (2.8)	22.5 (10.0)	32.2 (1.8)	35.5 (0.8)	26.4 (6.9)
Post-secondary Education	40.2 (8.4)	32.9 (3.0)	53.6 (22.7)	41.4 (6.3)	37.3 (3.4)	48.6 (18.4)
Not reported	3.5 (3.6)	1.7 (2.0)	6.8 (5.6)	4.0 (3.8)	2.1 (2.1)	7.3 (5.8)
Literacy level ^5^, % (SE)						
≤8th grade	36.6 (0.9)	47.7 (1.6)	16.1 (6.3)	29.1 (4.7)	37.1 (6.0)	15.1 (4.6)
>8th grade	62.3 (1.2)	51.6 (1.9)	82.0 (7.0)	70.1 (5.1)	62.0 (6.6)	84.3 (4.5)
Not reported	1.1 (0.5)	0.7 (0.4)	1.9 (0.7)	0.8 (0.5)	0.9 (0.7)	0.6 (0.5)
Lifestyle:						
Smoking status ^6^, % (SE)						
Currently smoking	49.8 (4.3)	56.4 (4.7)	37.7 (9.9)	35.8 (3.9)	38.4 (3.9)	31.3 (8.1)
Not currently smoking	47.9 (4.0)	40.8 (5.2)	61.0 (9.1)	61.3 (4.4)	58.3 (4.8)	66.6 (9.6)
Not reported	2.3 (1.0)	2.8 (1.1)	1.3 (1.0)	2.8 (1.1)	3.2 (1.7)	2.2 (1.6)
Self-reported health measures:						
Health status, % (SE)						
Excellent/very good	41.8 (3.0)	36.2 (1.6)	51.9 (10.6)	40.6 (3.8)	36.0 (2.6)	48.7 (9.1)
Good	40.4 (0.8)	45.8 (1.4)	30.6 (3.3)	40.1 (3.1)	43.1 (3.9)	35.0 (4.6)
Fair/poor	17.8 (2.4)	18.0 (1.7)	17.4 (7.4)	19.2 (2.7)	20.9 (2.7)	16.3 (4.8)
Menopause status, % (SE)						
Premenopausal	-	-	-	54.8 (1.5)	55.2 (3.1)	54.1 (2.8)
Postmenopausal	-	-	-	42.4 (2.9)	40.6 (5.3)	45.4 (2.4)
Not reported	-	-	-	2.8 (1.8)	4.1 (2.9)	0.5 (0.4)
Diagnostic health measures:						
Body mass index ^7^, mean (SE)	27.6 (0.2)	27.0 (0.2)	28.6 (0.2)	30.9 (0.6)	32.0 (0.7)	28.9 (1.7)
Metabolic syndrome ^8^, % (SE)	28.2 (2.2)	23.1 (4.7)	37.8 (5.2)	37.9 (5.6)	39.8 (3.5)	34.7 (11.2)
MetS risk factors: ^9^						
Elevated triglycerides ^10^, % (SE)	30.3 (4.9)	24.1 (5.2)	41.6 (7.2)	24.1 (2.7)	20.2 (2.8)	30.8 (5.2)
Low HDL-C ^11^, % (SE)	35.8 (1.8)	31.2 (3.6	44.3 (1.4)	48.7 (4.0)	50.9 (1.4)	44.9 (10.8)
Elevated blood glucose ^12^, % (SE)	30.2 (2.1)	28.5 (1.3)	33.5 (6.0)	26.2 (4.0)	26.7 (3.6)	25.2 (5.1)
Elevated blood pressure ^13^, % (SE)	40.6 (3.0)	40.2 (2.2)	41.4 (4.7)	44.2 (1.8)	51.9 (4.6)	30.7 (8.3)
Elevated waist circumference ^14^, % (SE)	34.1 (1.7)	29.4 (2.1)	42.7 (4.5)	71.0 (4.8)	77.3 (3.9)	60.0 (12.1)

^1^ Sample weights were applied to permit calculation of estimates representative of the Baltimore City African American and White adult (age 30–64 years) population. ^2^ Data collection for HANDLS Wave 1 took place from August 2004 to March 2009 [18]. ^3^ A stratification variable included in the HANDLS study design. Accordingly, estimates were adjusted during the calculation of sample weights. ^4^ Poverty status groups are based on the ratio of self-reported household income to the U.S. Department of Health and Human Services 2004 poverty guidelines [19], expressed as a percentage. ^5^ Literacy level was assessed using the Wide Range Achievement Test edition 3 (WRAT-3) reading subtest [21]. ^6^ Smoking status is based on cigarettes only. ^7^ Body mass index (BMI) = weight (kg)/height (m)^2^. ^8^ Metabolic syndrome is defined as the presence of ≥3 of the following risk factors: elevated triglycerides, low HDL-C, elevated blood glucose, elevated blood pressure, and elevated waist circumference [2]. ^9^ Triglycerides, HDL-C, and glucose were measured in whole blood collected after participants fasted for a minimum of 8 h [29]. ^10^ Elevated triglycerides: >150 mg/dL (1.7 mmol/L) and/or on lipid-lowering medication [2]. ^11^ HDL-C, High-density lipoprotein cholesterol. Low HDL-C: <40 mg/dL (1.0 mmol/L) for males, <50 mg/dL (1.3 mmol/L) for females and/or on lipid-lowering medication [2]. ^12^ Elevated blood glucose: fasting glucose ≥100 mg/dL (>5.6 mmol/L) and or on diabetes medication [2]. ^13^ Elevated blood pressure: systolic ≥130 mm Hg and/or diastolic ≥85 mm Hg and/or on blood pressure medication [2]. ^14^ Elevated waist circumference: ≥102 cm for males, ≥88 cm for females [2].

**Table 2:** Usual intakes ^1,2^ of total flavonoids, ^3^ mean and percentile distribution, by sex and race, HANDLS study, Wave 1 ^4^.

			Percentiles of Usual Intake						
		Mean	5th	10th	25th	50th	75th	90th	95th
	N	------------------------------------------------------- mg (SE) -------------------------------------------------							
Males:									
All	828	187.4 (10.6)	22.8 (3.6)	33.9 (4.3)	64.6 (6.0)	126.3 (8.9)	239.3 (13.5)	409.2 (26.4)	558.6 (43.4)
African American	480	172.7 (15.3)	20.8 (3.9)	31.5 (4.6)	60.4 (6.9)	117.1 (11.4)	219.6 (18.7)	377.4 (36.1)	510.5 (55.0)
White	348	214.5 (17.4)	27.1 (4.4)	39.5 (6.3)	75.8 (9.7)	147.2 (14.2)	276.9 (22.9)	467.5 (38.4)	632.5 (53.6)
Females:									
All	1009	255.2 (34.6)	19.5 (3.9)	31.9 (5.7)	68.5 (9.6)	149.8 (19.3)	315.6 (39.7)	588.2 (82.2)	837.8 (129.2)
African American	577	212.4 (37.4)	16.4 (3.0)	26.4 (4.4)	56.8 (8.0)	125.4 (18.7)	261.4 (44.4)	486.6 (87.4)	702.7 (139.3)
White	432	329.9 (35.9)	31.4 (8.5)	48.7 (11.0)	98.6 (16.5)	204.5 (24.5)	413.3 (42.7)	742.1 (83.1)	1053.7 (136.5)

^1^ Usual intake distributions were estimated via the NCI method [14]. ^2^ Sample weights were applied to permit calculation of estimates representative of the Baltimore City African American and White adult (30–64 years) population. ^3^ Defined as the summative total of anthocyanidins, flavan-3-ols (catechins, theaflavins, and thearubigins), flavanones, flavones, flavonols, and isoflavones [26]. ^4^ Data collection for HANDLS Wave 1 took place from August 2004 to March 2009 [18].

**Table 3:** Odds ^1^ of metabolic syndrome and its risk factors at the 75th versus 25th percentile of usual intake ^2,3,4^ of total flavonoids ^5^, HANDLS study, Wave 1 ^6^.

Metabolic Syndrome/Risk Factor ^7^	Males			Females		
	All	African American	White	All	African American	White
	-------------------------------------------------Odds ratio (95% CI)-------------------------------------------------					
Metabolic syndrome	0.62 (0.53, 0.71)	0.72 (0.53, 0.98)	0.72 (0.52, 0.98)	1.22 (0.92, 1.61)	1.24 (0.93, 1.65)	1.23 (0.93, 1.63)
Elevated triglycerides	0.86 (0.71, 1.04)	0.96 (0.74, 1.25)	0.96 (0.73, 1.26)	1.00 (0.81, 1.24)	1.15 (0.90, 1.48)	1.15 (0.90, 1.47)
Low HDL-C	0.52 (0.45, 0.61)	0.58 (0.42, 0.81)	0.57 (0.41, 0.81)	1.34 (1.07, 1.69)	1.36 (1.02, 1.82)	1.35 (1.02, 1.79)
Elevated blood glucose	0.79 (0.69, 0.89)	0.79 (0.72, 0.87)	0.79 (0.71, 0.87)	1.61 (1.18, 2.18)	1.57 (1.20, 2.06)	1.56 (1.19, 2.04)
Elevated blood pressure	0.78 (0.49, 1.24)	0.80 (0.51, 1.24)	0.79 (0.50, 1.25)	0.90 (0.68, 1.18)	0.79 (0.47, 1.33)	0.79 (0.47, 1.32)
Elevated waist circumference	1.85 (0.55, 6.19)	1.94 (0.69, 5.48)	1.98 (0.68, 5.74)	0.84 (0.50, 1.41)	0.81 (0.46 1.43)	0.81 (0.47, 1.43)

Abbreviations: CI, Confidence interval; HDL-C, high-density lipoprotein cholesterol. ^1^ Reflects exponentiated difference in log odds of metabolic syndrome/risk factor associated with a difference in usual total flavonoid intake between the 25th and 75th percentile values. Race (as appropriate), poverty status, age (as a continuous variable), education level, smoking status, literacy, menopause status (women only), health status, and body mass index were included in the logistic regression models. ^2^ Usual intake distributions were estimated using the NCI method [14]. ^3^ Sex-specific and sex-and-race-specific distributions were applied, as appropriate. ^4^ Sample weights were applied to permit calculation of estimates representative of the Baltimore City African American and White adult (30–64 years) population. ^5^ Defined as the summative total of anthocyanidins, flavan-3-ols (catechins, theaflavins, and thearubigins), flavanones, flavones, flavonols, and isoflavones [26]. ^6^ Data collection for HANDLS Wave 1 took place from August 2004 to March 2009 [18]. ^7^ Metabolic syndrome and its risk factors are defined by Alberti, K.G.M.M., et al. [2].

## Data Availability

The data presented in this study are available through collaboration agreements for the transfer of human data with the National Institute on Aging as described at https://handls.nih.gov/06Coll.htm (accessed on 16 February 2021). The FNDDS and Flavonoid Databases are available for download from the web pages in references [24,25], respectively.
